# Primary breast lymphoma in the right breast during treatment for left breast cancer

**DOI:** 10.1186/1477-7819-5-134

**Published:** 2007-11-26

**Authors:** Shigeyuki Nagata, Ataru Nishimura, Yukio Iwashita, Tadahiko Kinoshita, Kengo Fukuzawa, Hideya Tashiro, Kenzo Wakasugi

**Affiliations:** 1 Department of Surgery, Oita Red Cross Hospital, Oita, Japan

## Abstract

**Background:**

Primary breast lymphoma is a rare condition, and distinguishing it from breast cancer is important because their treatments differ radically. Moreover, a recent report showed that mastectomy offered no benefit in the treatment of primary breast lymphoma.

**Case presentation:**

A 59-year-old woman was treated with adjuvant chemotherapy and local radiation after surgery for left breast cancer. She presented with a rapidly growing mass in the right breast at 20 months after surgery. Mammography and computed tomography revealed a massive tumour. She was diagnosed with primary breast lymphoma by aspiration cytology, and surgery was performed. Histopathological and immunohistochemical findings confirmed a diffuse large B-cell type primary breast lymphoma.

**Conclusion:**

In this case, the lymphoma exhibited rapid growth despite chemotherapy for a malignancy in the contralateral breast. The patient had developed bronchiolitis obliterans organizing pneumonia due to radiation. Therefore, surgical treatment of the lymphoma was selected.

## Background

Primary breast lymphoma (PBL) is a rare condition, and it represents less than 0.7% of all malignant breast tumours. It affects less than 1% of patients with non-Hodgkin lymphoma (NHL) and constitutes only 2% of all extranodal NHLs [1-9]. Further, few reports are available on PBL, and the sample size used in all clinical studies is small.

The clinical presentation and radiological features of this condition do not differ from those of breast cancer. PBL is mostly observed in females. The most common presentation of either malignancy is a painless enlarging breast mass [1]. On a mammogram, these lymphomas may lack the irregular borders of infiltrating carcinomas, and more than half the tumors exhibit no calcification [1,2]. Despite the clinical and radiographic similarities between the 2 tumor types, their treatments differ radically. Hence, it is important to accurately distinguish lymphomas from other breast malignancies in order to appropriately triage patients for treatment. Fine-needle aspiration cytology is one of the most useful methods for the diagnosis of lymphoproliferative disorders, and its overall sensitivity is approximately 90% [2,3,7].

PBL treatment varies widely, and surgical therapy options range from biopsy to modified radical mastectomy. More recently chemotherapy using various agents has been recognized as the preferred treatment. Radiotherapy may be used as an adjuvant therapy or as a primary local therapy [9,10]. Treatment options may also include immunotherapy or radioimmunotherapy. A very recent study reported that mastectomy offered no benefit in the treatment of PBL, and treatment that included chemotherapy and/or radiation therapy provided benefits with regard to both the survival and recurrence rates [11].

With such paucity of data, it is more difficult to derive valid conclusions regarding the epidemiology, appropriate management, and prognosis of PBL as compared to lymphomas at other sites. Many reports conclude that the breast is an unfavourable primary site and that this malignancy exhibits a significantly poorer prognosis than other localized lymphomas [4,8]. However, no clear consensus for therapy has emerged. Therefore, accumulation of data in the form of case reports, clinical studies, etc., is important.

In this study, we report the case of a female patient who underwent treatment for breast cancer and later presented with a rapidly growing PBL in the contralateral breast. This is an extremely unusual case because two considerably different diseases were manifested in single patient. Selecting the best treatment option from among chemotherapy, radiation, surgical therapy, etc was difficult. This report describes and discusses the physical findings, diagnosis, and treatment of this condition.

## Case presentation

**Figure 1:**
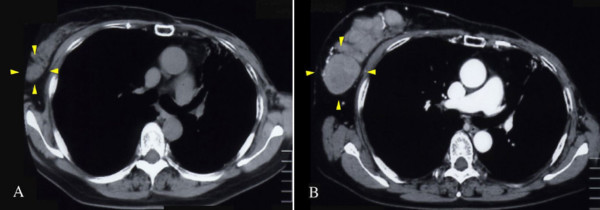
**Chest CT images**. A) CT was performed on November 22, 2005. B) CT was performed on December 21, 2005. The tumour apparently enlarged during a month. The doubling time was calculated at the same area (arrow) of the tumour.

**Figure 2:**
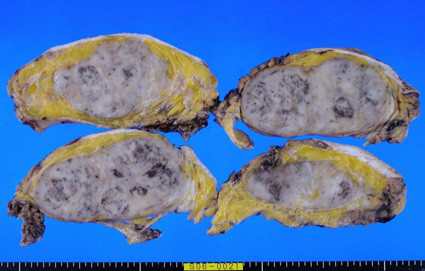
**Macroscopic findings of the tumour**. The resected tumour appeared aggregated with some nodules.

**Figure 3:**
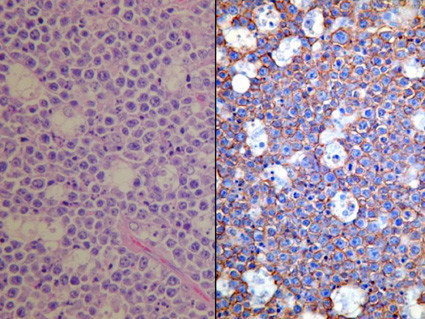
**Histological findings of the tumour**. The tumour was stained by hematoxylin and eosin (left, ×400) and showed positive reaction to L26 (right, ×400).

A 58-year-old woman underwent total mastectomy and dissection of the axillary lymph nodes (Auchincloss-Madden method) as treatment for left breast cancer in April 2004. Based on histological findings, she was diagnosed with stage IIB invasive ductal carcinoma (T2 N1 M0), and immunohistochemical findings revealed that the tumour cells were strongly positive for HER-2, very weakly positive for the oestrogen receptor, and negative for the progesterone receptor. The patient was sequentially treated with adjuvant chemotherapy (5 cycles of pirarubicin and cyclophosphamide [AC therapy; total dose of pirarubicin, 250 mg]). After AC therapy, she was administered tegafur/uracil (UFT; 400 mg/day). In March 2005, she complained of reddish skin eruptions over the left chest. Biopsy revealed a diagnosis of cancer recurrence, and she was treated with local radiation (total dose, 60 Gy). The areas of reddish skin eruptions apparently decreased, and chemotherapy (90 mg/body paclitaxel every week) was administered after radiation therapy. Later, the patient presented with high-grade fever, and a diffuse infiltrative shadow was seen on a chest X-ray image in October 2005. Based on her past history and chest computed tomography (CT) findings, she was diagnosed with bronchiolitis obliterans organizing pneumonia (BOOP) due to radiation. She was consequently treated with oral prednisolone (starting dose, 40 mg/day; maintenance dose, 15 mg/day) for 2 months. Her symptoms and imaging findings indicated improvement. In November 2005, a mass (diameter, <5 cm) in the right breast was recognized without any other symptoms. After a month, the huge mass in the right breast could be palpated; it was >10 cm in diameter, distended, and slightly tender. The laboratory blood levels of almost all test items were within the normal range, except for the level of lactate dehydrogenase (LDH) that was 873 IU/L (normal range, 200–400 IU/L). The tumour marker levels were as follows: carcinoembryonic antigen (CEA), 1.6 ng/mL (normal range, <5.0 ng/mL); carbohydrate antigen 15-3 (CA15-3), 8.0 U/mL (normal range, <31 U/mL); and soluble interleukin-2 receptor (sIL-2R), 1390 U/mL (normal range, 220–530 U/mL). A mammogram revealed a large lobulated tumour lodged in the right mammary gland with no microcalcifications. Ultrasound (US) demonstrated that the lesion comprised well-defined hypoechoic nodules with low-level internal echoes and that it enhanced through transmission; these findings were consistent with those of a malignant lymphoma. Ga-67 scintigraphy showed intense accumulation in the tumour. These findings were supported by those of fine-needle aspiration cytology that revealed large dysplastic lymphocytes with irregular nuclei and numerous mitotic figures. Chest CT revealed multiple nodules in the right breast that were uniformly enhanced and had a density identical to the soft tissue density; no apparent axillary lymph node swelling was noted. This tumour showed rapid growth, and its doubling time was calculated to be 8 days (Figure 1). Because this patient had undergone chemotherapy for breast cancer and had BOOP due to radiation, we considered it difficult for her to undergo additional chemotherapy or radiation therapy for the breast lymphoma; the patient finally opted for surgical therapy. Total mastectomy and axillary dissection (Auchincloss-Madden method) were performed in January 2006 (Figure 2). Based on the morphologic features of the tumour and immunophenotyping, the PBL was diagnosed to be of the diffuse large B-cell type (World Health Organization classification). The tumour cells were negative for CD3 and were diffusely positive on staining for the leukocyte common antigen L26, CD10, and bcl-2 (Figure 3). The level I lymph nodes were infiltrated by lymphoma cells. The patient received 8 cycles of chemotherapy (rituximab, 600 mg/body and CHOP [cyclophosphamide, 700 mg/body; doxorubicin, 45 mg/body; vincristine, 3 mg/body; and methylprednisolone, 250 mg/body] every 4 weeks). She was clinically disease free at 1 year after surgery.

## Discussion

The risk of secondary malignancies among female breast cancer patients has been studied for decades. Yu G *et al*., reported that the risk ratio of NHL assessed by comparing female breast cancer patients to the general population was 2.1 in the young group (20–49 years) [12].

The most common type of PBL is the diffuse large B-cell type, and its characteristics are consistent with those observed in the present case [1,2,7]. For PBL diagnosis, most studies use the criteria described by Wiseman and Liao: (1) A pathological specimen that demonstrates a close association between the lymphomatous infiltrate and the breast tissue is available in sufficient amount. (2) There is no evidence of concurrent widespread disease or preceding extramammary lymphoma. (3) The breast is the clinical site of presentation, but ipsilateral lymph node involvement is considered acceptable if both lesions develop simultaneously [3].

In middle-aged females, PBLs are most commonly detected as a solitary palpable mass. Men are rarely affected by this disease. Many studies report that PBL is more common in the right breast, especially the upper outer quadrant, which is almost consistent with the findings in this case [1,7].

PBLs are not usually detected by screening mammography. However, mammograms obtained after the identification of a palpable mass demonstrate a parenchymal abnormality in most cases. There is no specific mammographic characteristic that can be used for lymphoma diagnosis. The most common mammographic findings include a solitary well-defined mass that may have an irregular border, which is consistent with the finding in the present case. PBLs rarely demonstrate calcification or a spiculated appearance on mammography [1,2,5,7].

US evaluation of PBL usually demonstrates a hypoechoic lesion with well-defined borders that lack significant posterior enhancement or acoustic shadowing that may falsely indicate a benign cyst; these findings are similar to those in this case [1,2,5]. However, they are not a consistent feature of PBL because other reports have mentioned many variations [13].

Thus far, no clear clinical or radiological features that can distinguish PBLs from infiltrating breast carcinomas have been found. Adequate tissue biopsy based on histopathological evaluation and immunophenotyping continues to be the key in evaluating these patients [1-3,6,7].

There are various treatment options for PBL: surgery, radiation, and chemotherapy have been used alone or as combination therapy. Chemotherapy with or without adjuvant radiotherapy is the most common therapeutic approach [1-3,9,10]. The role of surgery in PBL treatment is often restricted to obtaining adequate tissue for accurate lymphoma diagnosis and classification, and PBL treatment is similar to that for lymphomas at other sites [1,3]. A recent paper showed that treatment by mastectomy offered no survival benefit or protection from recurrence [11]. It reported that treatment including radiation therapy in node-negative patients and treatment including chemotherapy in node-positive patients exhibited benefit with regard to both survival and recurrence rates.

The survival rates of patients with PBL are comparable to those with lymphomas in general and are favorable when compared to the survival rates of those with breast carcinoma. Prognosis depends on the histological tumor grade [1,4,8]. The international Prognostic Index considers age, LDH levels, performance status, Ann Arbor staging, and the presence of extranodal tumor in predicting the 5-year survival [2,14]. The most common site of relapse is reportedly the central nervous system [8-10].

This patient showed an unusual clinical course: PBL was discovered 20 months after surgery for a malignancy in the opposite breast. The tumour was recognized after the patient was treated with adjuvant AC chemotherapy and radiation therapy for local recurrence of breast cancer and while she was receiving oral prednisolone for BOOP. The patient was administered 3 of the 4 drugs used in CHOP chemotherapy for malignant lymphoma; despite this, PBL developed. Finally, due to the rapid growth of the tumour, resection was performed instead of chemotherapy or radiation therapy.

## Conclusion

It is difficult to select the treatment modality for PBL, but we considered surgical therapy to be better in this very special case. The patient was pronounced disease free at 1 year after surgery.

## Competing interests

The author(s) declare that they have no competing interests.

## Authors' contributions

**SN **conceived the study, participated in its design and coordination, and drafted the manuscript.

**AN **carried out the literature search and helped in drafting the manuscript.

**YI **and **TK **helped in drafting the manuscript.

**KF, HT**, and **KW **shaped the idea for the study and coordinated the study.

All authors have read and approved the final manuscript.
